# Development and Characterization of *Inula britannica* Extract-Loaded Liposomes: Potential as Anti-Inflammatory Functional Food Ingredients

**DOI:** 10.3390/antiox12081636

**Published:** 2023-08-18

**Authors:** Chi Rac Hong, Eun Ha Lee, Young Hoon Jung, Ju-Hoon Lee, Hyun-Dong Paik, Sung-Chul Hong, Seung Jun Choi

**Affiliations:** 1 Department of Food Science and Technology, Seoul National University of Science and Technology, Seoul 01811, Republic of Korea; hongcr@seoultech.ac.kr; 2 Smart Farm Research Center, Korea Institute of Science and Technology, Gangneung 25451, Republic of Korea; ehlee@kist.re.kr; 3 School of Food Science and Biotechnology, Kyungpook National University, Daegu 41566, Republic of Korea; younghoonjung@knu.ac.kr; 4 Department of Food and Animal Biotechnology, Research Institute of Agriculture and Life Science, Seoul National University, Seoul 08826, Republic of Korea; juhlee@snu.ac.kr; 5 Department of Food Science and Biotechnology of Animal Resources, Konkuk University, Seoul 05029, Republic of Korea; hdpaik@konkuk.ac.kr; 6 Department of Food Science and Biotechnology, Kunsan National University, Gunsan 54150, Republic of Korea; 7 Center for Functional Biomaterials, Seoul National University of Science and Technology, Seoul 01811, Republic of Korea

**Keywords:** liposomes, *Inula britannica*, nanocarrier, anti-inflammation, RAW 264.7 macrophage cell

## Abstract

We investigated the potential of *Inula britannica* extract encapsulated in liposomes as a functional food ingredient with enhanced bioavailability and stability. *Inula britannica*, known for its anti-inflammatory properties and various health benefits, was encapsulated using a liposome mass production manufacturing method, and the physical properties of liposomes were evaluated. The liposomes exhibited improved anti-inflammatory effects in lipopolysaccharide-activated RAW 264.7 macrophages, suppressing the production of pro-inflammatory mediators such as nitric oxide and prostaglandin E2 and downregulating the expression of iNOS and COX-2 transcription factors. Additionally, we observed reduced production of pro-inflammatory cytokines TNF-α, IL-6, and IL-1β, and modulation of the NF-κB and mitogen-activated protein kinase signaling pathways. These findings suggest that *Inula britannica* extract encapsulated in liposomes could serve as a valuable functional food ingredient for managing and preventing inflammation-related disorders, making it a promising candidate for incorporation into various functional food products. The enhanced absorption and stability provided by liposomal encapsulation can enable better utilization of the extract’s beneficial properties, promoting overall health and well-being.

## 1. Introduction

Medicinal herbs, such as those traditionally used in the Republic of Korea, Japan, and China, have long been studied for their potential as natural remedies for health and disease prevention [1]. Although these herbs have been developed into pharmaceuticals, they are often studied for their potential as functional food ingredients due to their mild efficacy and potential for long-term consumption without side effects [2,3]. One such herb is *Inula britannica* L. (*I. britannica*), which is commonly consumed as a tea or in combination with honey in East Asia and is known to have anti-inflammatory properties that can prevent respiratory conditions such as asthma and bronchitis [4,5,6]. Additionally, *I. britannica* extract has been reported to improve liver function, protect nerve cells in the brain, and prevent diabetes, among other beneficial effects [5,7,8,9]. *I. britannica* extract has also been used in cosmetics for its anti-wrinkle and skin-whitening effects owing to its anti-oxidative properties [10,11].

*I. britannica* extract contains various phytochemicals, including sesquiterpene lactones, triterpenoids, and flavonoids, which possibly contribute to its various physiological effects [4]. The sesquiterpene lactones in the extract include gaillardin, britannin, 11,13-dihydroinuchinenolide B, ivalin, and pulchellin C, while the triterpenoids consist of 3-O-palmitates of 16β-hydroxylupeol, 16β-hydroxy-β-amyrin, and faradiol [12]. Additionally, the extract contains flavonoids such as quercetin, luteolin, and luteolin-7-O-glucoside [13]. These phytochemicals have been reported to exhibit anti-microbial effects against *Salmonella*, *Helicobacter pylori*, and *Staphylococcus aureus* and anti-tumor effects via regulation of the Bcl-2 family in ovarian, breast, and prostate cancer cells [14,15,16]. *I. britannica* extract also prevents Alzheimer’s disease, reduces inflammation in mast cells, and prevents skin aging [17,18].

These antioxidant-rich phytochemicals in *I. britannica* extract demonstrate intricate interplay within the mechanisms of intracellular oxidation and inflammation [4]. Reactive oxygen species (ROS) are generated in the normal metabolic process, but if excessively increased, they induce oxidative stress that damages cellular components [19]. This oxidative stress impairs DNA, proteins, and fatty acids, serving as the underlying cause of various diseases such as inflammation, cancer, and cardiovascular diseases [20]. Therefore, antioxidants neutralize ROS and reduce such oxidative stress, thereby helping to prevent or alleviate the onset of inflammation and related diseases [19,21]. Additionally, the two intertwined mechanisms show that oxidative stress promotes inflammatory responses, and inversely, inflammation increases the production of ROS [22,23]. Hence, antioxidants can aid in reducing inflammation, and anti-inflammatory substances can help mitigate oxidative stress. Simultaneously regulating these two mechanisms can play a significant role in preventing and treating many diseases.

Despite the many benefits of phytochemicals as functional food ingredients, their highly unsaturated (double-bonded) structural characteristics make them vulnerable to oxidation and degradation in external environments, reducing their usefulness in improving physiological effects before absorption in the body [24]. Poor solubility in aqueous solutions also limits their bioavailability [25]. Encapsulation with nanocarriers, such as liposomes, is a strategy for achieving the physical and biological stability necessary to maximize the potential physiological effects of phytochemicals [26]. Liposomes are colloidal structures composed of amphiphilic phospholipids that can encapsulate hydrophilic, lipophilic, and amphiphilic compounds. They exhibit significant effects on improving the physiological activity of core materials by mimicking cell membranes [27].

In particular, nano-liposomes can improve the bioavailability of phytochemicals by increasing the rate and extent of absorption owing to their small size [26]. However, a major obstacle to the application of nanotechnology is the challenge of mass production. While lab-scale devices, such as microchips, have been used to manufacture nanocarriers such as liposomes, studies on mass production and the resulting quality and physiological effects of liposomes are limited [28]. Therefore, we aimed to evaluate the physical properties of liposomes and investigate the improved physiological effects of an encapsulated vulnerable extract by using a high-purity liposome manufacturing method for mass production.

In this study, we prepared nano-liposomes containing a vulnerable extract, a compound known to exhibit various physiological effects such as anti-oxidative, anti-inflammatory, and anti-tumor effects. We aimed to confirm the degree of improvement in physiological effects by encapsulating the *I. britannica* extract in nano-liposomes to enhance its absorption and stability in the body.

## 2. Materials and Methods

### 2.1. Preparation of I. britannica Aqueous Extract

Dried *I. britannica* powder was purchased from Kyungdong market (Seoul, Republic of Korea) and stored at 4 °C. Subsequently, 100 g of dried *I. britannica* powder was mixed with 1000 mL of distilled water and extracted at 60 °C for 48 h. The extract was filtered using Whatman No. 2 paper (GE Healthcare, Chicago, IL, USA) to remove debris. The filtered solution was then concentrated by removing a portion of the solvent using a rotary evaporator (Eyela, Tokyo, Japan). The concentrated extract was stored in a deep freezer at −80 °C for 12 h, freeze-dried, and stored at 4 °C.

### 2.2. Preparation of Encapsulated I. britannica with Nano-Liposome

We prepared the nano-liposome by adapting and modifying the method originally proposed by Yang et al. [29]. A total of 1 g of lecithin (ES Foods, Gyunggi, Republic of Korea) and 0.125 g of β-sitosterol (Acros, Morris Palins, NJ, USA) were dissolved in a 1:3 mixture (200 mL) of *n*-hexane (J.T. Baker, Phillipsburg, NJ, USA) and ethyl acetate (J.T. Baker). Additionally, the *I. britannica* extract was dissolved in 100 mL of distilled water at 10 mg/mL. Then, these two solutions were mixed and stirred at 15,000 RPM for 1 min to produce a reverse micelle solution using a high-speed blender (ULTRA-TURRAX, IKA, Staufen, Germany). The reverse micelle solution was then homogenized using a microfluidizer (M110EH Microfluidizer, Microfluidics, Westwood, MA, USA) at 1000 bar for 10 repetitions. The homogenized reverse micelle solution (100 mL) and *I. britannica* extract (10 mg/mL in distilled water, double volume) were mixed for 1 min at 15,000 RPM in a high-speed blender, followed by 5 repetitions at 500 bar in a microfluidizer to form a phospholipid bilayer. The solvents were partially removed from the liposome solution using a rotary evaporator, and then the liposome solution was stored at 4 °C in a dark, refrigerated room.

### 2.3. Characterization of Encapsulated I. britannica with Nano-Liposome

#### 2.3.1. Analysis of Particle Size and Stability

The dynamic light scattering (DLS) technique was applied using a Zetasizer Nano ZS system (DTS1070, Malvern Instruments Ltd., Malvern, UK) to evaluate the particle size and polydispersity index (PdI) of liposomes. The sample was analyzed with a refractive index of 1.330, a viscosity of 0.8872 Cp, an equilibration time of 1 min, a temperature of 25 °C, and an angle of backscattering measurement of 173°. The stability of the liposomes was assessed by measuring their zeta potential. Three samples were injected into disposable capillary cells (DTS1070, Malvern Instruments Ltd.) at 25 °C.

#### 2.3.2. Morphological Analysis of Nano-Liposome Encapsulating *I. britannica* Extract

The morphology of the produced liposomes was observed using a transmission electron microscope (TEM, 120 keV, JEOL Ltd., Tokyo, Japan). The liposomes were stained using a 2% (*w*/*v*) solution of uranyl acetate to enhance their visibility under the TEM. After depositing 10 µL of the sample on a 200-mesh grid, the staining reagent was applied and allowed to sit for a minute. Subsequently, the grid was rinsed with tertiary distilled water. Once the grids were fully dried at a temperature of 25 °C, they were inspected using the TEM.

### 2.4. Purification of Encapsulated I. britannica with Nano-Liposome

The *I. britannica* extract, both in its nano-liposome-encapsulated form and non-encapsulated form, was individually separated and purified using a pre-packed Sephadex G-25 column (PD-10, GE Healthcare, Little Chalfont, UK). The column was first equilibrated with distilled water as the eluent. The retentate was then loaded onto the column in a volume of 2.0 mL, with 0.5 mL aliquots subsequently collected. The initial 2.0 mL of the eluent was discarded, while the subsequent 3.5 mL was collected to determine encapsulation efficiency. After purification, the purity of the resulting samples was analyzed using high-performance liquid chromatography (HPLC).

### 2.5. Determination of Encapsulation Efficiency by Measuring Chlorogenic Acid

#### 2.5.1. Quantification of Chlorogenic Acid in *I. britannica* Extract by High-Performance Liquid Chromatography (HPLC)

The chlorogenic acid content in the *I. britannica* extract was quantified using an Agilent 1260 Infinity Liquid Chromatograph (Agilent Technologies, Santa Clara, CA, USA). This system was equipped with a quaternary pump delivery system (G1331B), an auto-sampler (G1329B), a column thermostat (G1316A), and a diode array and multiple wavelength detector (G1315D). A volume of 10 µL from each pre-treated sample was injected into an Eclipse XDB-C18 column (4.6 × 150 mm, 5 µm, Agilent Technologies Inc., Santa Clara, CA, USA) set at 35 °C, and detection was carried out at λ = 330 nm. The mobile phase was composed of water (J.T. Baker) and acetonitrile (J.T. Baker), both containing 0.1% (*v*/*v*) formic acid (Sigma-Aldrich, Saint Louis, MO, USA), which served as eluents A and B, respectively. The separation was executed at a flow rate of 0.8 mL/min using a gradient program. The gradient was programmed as follows:

- 0 to 5 min, 10 to 20% B;
- 5 to 10 min, maintaining 20% B;
- 10 to 15 min, 20 to 30% B;
- 15 to 25 min, 30 to 80% B;
- 25 to 28 min, 80 to 10% B;
- 28 to 30 min, holding at 10% B;

The analysis ran for a total of 30 min.

#### 2.5.2. Encapsulation Efficiency

The encapsulation efficiency of the *I. britannica* extract was calculated using the following formula:

$$\mathrm{Encapsulation}\;\mathrm{Efficiency}\;\left(\%\right)=\frac{\mathrm{Total}\;\mathrm{Material}\;-\;\mathrm{Free}\;\mathrm{Material}}{\mathrm{Total}\;\mathrm{Material}}\times 100.$$

First, the sample was loaded into an ultrafiltration tube and centrifuged for 10 min at 4000× *g*. Second, the volumes of the collected retentates and filtrates were measured, and the chlorogenic acid concentration in each was evaluated using HPLC after Triton-X 100 (Sigma-Aldrich) treatment, as described earlier (Section 2.5.1). Finally, the encapsulation efficiency was calculated using the formula mentioned above, which considers the amount of chlorogenic acid in both the retentate and filtrate as well as the amount of free material present in the filtrate.

### 2.6. Cell Culture

RAW 264.7 cells, obtained from the American Type Culture Collection (Rockville, MD, USA), were cultured in Dulbecco’s Modified Eagle’s Medium supplied by HyClone (Logan, UT, USA). The medium was supplemented with 10% fetal bovine serum and 1% penicillin and streptomycin. The cells were maintained at 37 °C in an incubator with 5% CO_2_.

### 2.7. Cytotoxicity

The cytotoxicity of the *I. britannica* extract and the *I. britannica* extract encapsulated in liposomes was evaluated using an MTT assay. To conduct the MTT assay, 5 × 10^4^ RAW264.7 cells per well were seeded in 96-well plates and pre-cultured for 24 h. The cells were then exposed to 1 µg/mL lipopolysaccharide (LPS, Sigma-Aldrich), the *I. britannica* extract, and the *I. britannica* extract, encapsulated in liposomes, and incubated for an additional 24 h. The cells were treated with 20 µL of MTT solution (3-(4,5-dimethylthiazol-2-yl)-2,5-diphenyltetrazolium bromide, Sigma-Aldrich) for 4. Formazan crystals were dissolved in 100 µL of dimethyl sulfoxide, and the absorbance at 570 nm was measured using a microplate reader (Thermo Fisher Scientific, Waltham, MA, USA). Cell viability was calculated as a percentage relative to the control group.

### 2.8. Measurement of Nitric Oxide (NO) Production

The NO concentration in the culture medium was assessed using the Griess reaction assay. RAW 264.7 cells were seeded at a density of 5 × 10^5^ cells per well in a 96-well plate. The seeded cells were pre-treated with various concentrations of *I. britannica* extract encapsulated in liposomes for two hours. Following pre-treatment, the cells were stimulated with 1 µg/mL lipopolysaccharide (LPS, Sigma-Aldrich) and incubated for 24 h at 37 °C in a 5% CO_2_ environment. Following incubation, the Griess reagent was mixed 1:1 with 100 µL of culture medium and allowed to react for 15 min at room temperature. The absorbance was then measured at 540 nm using a microplate reader. The amount of NO produced was quantified by comparison with a standard curve generated from various concentrations of sodium nitrite.

### 2.9. Prostaglandin E2-Enzyme-Linked Immunosorbent Assay (PGE_2_-ELISA)

The cells were pre-treated with *I. britannica* extract and *I. britannica* extract encapsulated in liposomes for 2 h, under the same conditions as the NO production assay, then incubated with LPS (1 μg/mL) for 24 h at 37 °C in a 5% CO_2_ environment. The anti-inflammatory activity of the extract and liposomes was evaluated by determining the amount of PGE2 in the culture medium by measuring the absorbance at 405 nm using the PGE2-ELISA kit from R&D Systems (Minneapolis, MN, USA), following the manufacturer’s instructions.

### 2.10. Measurement of Cytokine Expression Using Quantitative Real-Time Polymerase Chain Reaction (qRT-PCR)

RAW 264.7 cells were seeded at a density of 1 × 10^6^ cells per well in 6-well plates and allowed to adhere for 24 h. These cells were then pre-treated with varying concentrations of *I. britannica* extract encapsulated in liposomes for 2 h, followed by stimulation with LPS (1 µg/mL) for an additional 24 h at 37 °C in a 5% CO_2_ environment. Total RNA was extracted using the RNeasy Mini Kit (Qiagen, Hilden, Germany), and 2 µg of the total RNA was reverse transcribed to cDNA using the RevertAid™ First Strand cDNA Synthesis Kit (Thermo Fisher Scientific). The expression levels of anti-inflammatory cytokines were evaluated using semi-quantitative real-time PCR (PikoReal 96, Thermo Fisher Scientific) and SYBR Green PCR Master Mix (Thermo Fisher Scientific). The primers used in the experiment were the genes of TNF-α (forward: 5′-TTGACCTCAGCGCTG AGTTG-3′ and reverse: 5′-CCTGTAGCCCACGTCGTAGC-3′), IL-6 (forward: 5′-GTACTCCAGAAGACC AGAGG-3′ and reverse: 5′-TGCTGGTGACAACCACGGCC-3′), IL-1β (forward: 5′-CAGGATGAGGACATGAGCACC-3′ and reverse: 5′-CTCTGCAGACTCAAACTCCAC-3′), iNOS (forward: 5′-CCCTTCCGAAGTTT CTGGCAGCAGC-3′ and reverse: 5′-GGCTGTCAGAGCCTCGTGGCTTTGG-3′), COX-2 (forward: 5′-CACT ACATCCTGACCCACTT-3′ and reverse: 5′-ATGCTCCTGCTTGAGTATGT-3′), and β-actin (forward: 5′-GTG GGCCGCCCTAGGCACCAG-3′ and reverse: 5′-GGAGGAAGAGGATGCGGCAGT-3′). The PCR protocol consisted of an initial activation step at 95 °C for 2 min, followed by 40 cycles of denaturation at 95 °C for 5 s and annealing and extension at 60 °C for 15 s. The delta-delta CT method was used to analyze the results, and a melting curve analysis was performed to confirm reaction specificity.

### 2.11. Western Blot

To analyze NF-κB and mitogen-activated protein kinase (MAPK) phosphorylation, RAW264.7 cells were seeded at a density of 5 × 10^5^ cells per well in a 6-well plate and pre-cultured for 24 h. These cells were then pre-treated for two hours with various concentrations of *I. britannica* extract encapsulated in liposomes, followed by stimulation with LPS (1 µg/mL) for 24 h at 37 °C in a 5% CO_2_ environment. After washing the cells with cold phosphate-buffered saline, they were lysed using a radioimmunoprecipitation assay buffer supplemented with protease and phosphatase inhibitors. The lysates were then subjected to a freeze-thaw cycle for 2 min in an ice bath, followed by sonication in three cycles (5 s pulse on and 5 s pulse off). The sonicated samples were centrifuged at 14,000× *g* for 20 min at 4 °C. The supernatant’s total protein content was assessed using the DCTM Protein Assay Kit (Bio-Rad, Hercules, CA, USA). Next, a total of 30 µg of each lysate was separated on 8–12% sodium dodecyl sulfate-polyacrylamide gel electrophoresis, transferred to a polyvinylidene fluoride membrane, and blocked with tris-buffered saline containing 5% skim milk and Tween 20 for 2 h to prevent non-specific binding. The membrane was then incubated with the primary antibody diluted 1:5000 at 4 °C overnight and with the secondary antibody at room temperature for 2 h. Bands were detected using the ECL system (Bio-Rad), and images were captured on an X-ray film. The intensity of the bands was quantified using Image J software (National Institutes of Health, Bethesda, MD, USA) [30].

### 2.12. Statistical Analysis

Data for each experiment were collected from three independent trials, and the mean and standard deviation were calculated for each set of results. The statistical significance of the findings was assessed using an analysis of variance (ANOVA), and differences between means were evaluated using Tukey’s post hoc test (*p* < 0.05 was considered statistically significant). All statistical analyses were conducted using IBM SPSS Statistics software, version 18 (IBM Inc., New York, NY, USA).

## 3. Results

### 3.1. Characterization of Liposomes Encapsulating I. britannica Extract

**Figure 1:**
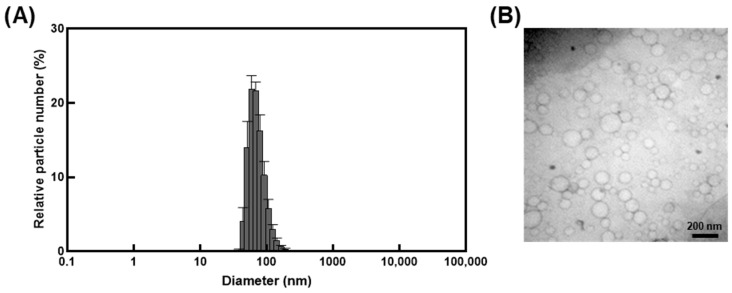
Characterization of liposomes encapsulating *I. britannica* extract. (**A**) The size distribution of liposomes encapsulating *I. britannica* extract. (**B**) Transmission electron microscopic analysis of liposomes encapsulating *I. britannica* extract.

Encapsulation of *I. britannica* extract within nanocarrier liposomes indicated that the liposomes had a narrow size distribution with an average diameter of 101.3 ± 5.6 nm and a PdI of less than 0.2 (Figure 1A and Table 1). The zeta potential indicated an absolute value of 42.2 ± 0.7 mV (Table 1), exhibiting stable dispersion of the liposomes in solution. The TEM image confirmed the spherical shape and size (less than 200 nm) and the morphological characteristics of the liposome particles (Figure 1B).

### 3.2. Encapsulation Efficiency of I. britannica Extract in Liposomes

To assess the encapsulation efficiency of *I. britannica* extract in liposomes, we performed an HPLC analysis using chlorogenic acid as an internal standard, primarily owing to its high abundance in the *I. britannica* extract (Figure S1). The encapsulation efficiency was found to be 17.28 ± 0.39%.

### 3.3. Effect of I. britannica Extract Encapsulated in Liposomes on LPS-Induced Inflammation in RAW 264.7 Cells

**Figure 2:**
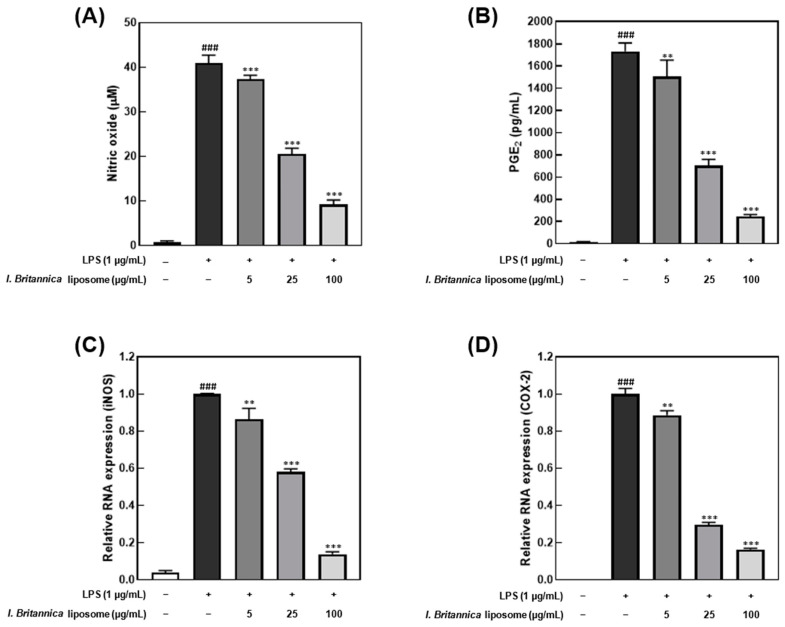
Anti-inflammatory effects of *I. britannica* extract encapsulated in liposomes in lipopolysaccharide (LPS)-induced RAW 264.7 cells. (**A**) Nitric oxide (NO) production (μM); (**B**) prostaglandin E2 (PGE_2_) production (pg/mL); the relative intensity of (**C**) iNOS; and (**D**) COX-2 expression. Data are represented as mean ± SEM (error bar). ^###^ *p* < 0.001 versus non-LPS-induced cells. ** *p* < 0.01, and *** *p* < 0.001 versus LPS-only treated cells. The data were statistically analyzed using a one-way ANOVA followed by Tukey’s post hoc test.

The *I. britannica* extract encapsulated in liposomes exhibited inhibitory effects on NO and PGE_2_ production in LPS-stimulated RAW 264.7 cells. The cell viability treated with the *I. britannica* extract and the *I. britannica* extract encapsulated in liposomes was assessed using the MTT assay, and both extracts were found to be non-toxic at the tested concentrations (Figure S2). Furthermore, treatment with 100 μg/mL of *I. britannica* extract encapsulated in liposomes resulted in a significant reduction in NO production, by 77.50% (40.97 μM), compared with that of cells treated with only LPS (Figure 2A). The suppression of PGE_2_ synthesis by *I. britannica* extract encapsulated in liposomes was concentration-dependent, with the highest liposome concentration of 100 μg/mL reducing PGE_2_ production to 85.95% (243.41 pg/mL) (Figure 2B). Furthermore, qRT-PCR analysis confirmed that *I. britannica* extract encapsulated in liposomes dose-dependently decreased the expression of iNOS and COX-2 genes in LPS-stimulated RAW 264.7 cells. Particularly, iNOS expression was significantly increased in the LPS-treated group but was significantly decreased in a dose-dependent manner in cell lines treated with 5, 25, and 100 μg/mL of *I. britannica* extract encapsulated in liposomes (Figure 2C). Similarly, PGE_2_ production is regulated by COX-2 gene expression, and *I. britannica* extract encapsulated in liposomes dose-dependently decreased COX-2 gene expression. As a result, COX-2 gene expression significantly increased in the LPS-treated group but significantly decreased in a dose-dependent manner in the cell lines treated with 5, 25, and 100 μg/mL of *I. britannica* extract encapsulated in liposomes (Figure 2D).

### 3.4. Effect of I. britannica Extract Encapsulated in Liposomes on Pro-Inflammatory Cytokine Production and mRNA Expression in LPS-Induced RAW 264.7 Cells

**Figure 3:**
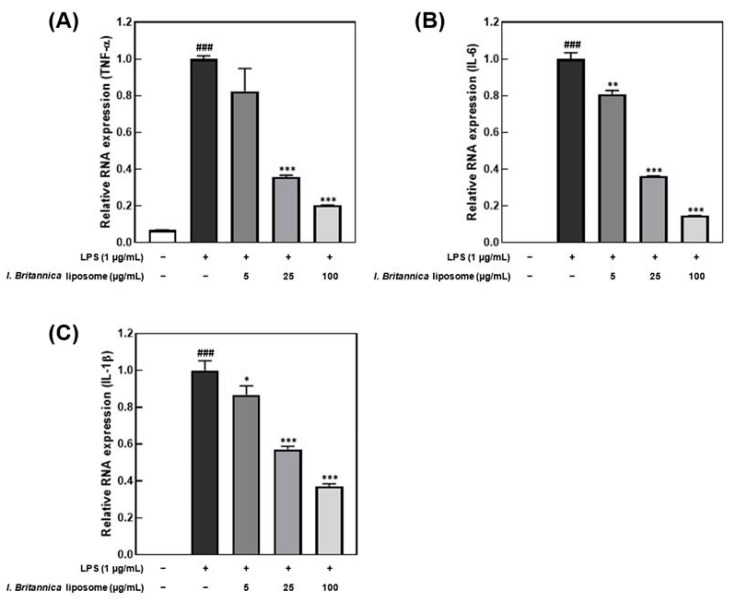
Effects of *I. britannica* extract encapsulated in liposomes on the mRNA expression of proinflammatory cytokines in lipopolysaccharide (LPS)-induced RAW 264.7 cells. (**A**) TNF-α; (**B**) IL-6; and (**C**) IL-1β expression. Data are represented as mean ± SEM (error bar). ^###^ *p* < 0.001 versus non-LPS-induced cells. * *p* < 0.05, ** *p* < 0.01, and *** *p* < 0.001 versus LPS-only treated cells. The data were statistically analyzed using a one-way ANOVA followed by Tukey’s post hoc test.

Lipopolysaccharide promotes the production of inflammatory cytokines, such as interleukin and TNF-α, in macrophages [31]. In this study, an inflammatory response was induced in macrophages by treatment with 1 μg/mL LPS. We investigated the effect of *I. britannica* extract encapsulated in liposomes on the reduction of pro-inflammatory cytokines. Treatment with *I. britannica* extract encapsulated in liposomes dose-dependently reduced the expression of TNF-α, IL-6, and IL-1β. Treatment with 5, 25, and 100 μg/mL of *I. britannica* extract encapsulated in liposomes reduced TNF-α expression by 17.56 ± 12.48%, 64.15 ± 0.87%, and 79.56 ± 0.07%, respectively, compared with that of the LPS-treated group (Figure 3). Similarly, treatment with 5, 25, and 100 μg/mL of *I. britannica* extract encapsulated in liposomes reduced IL-6 expression by 19.08 ± 1.96%, 63.80 ± 0.13%, and 85.35 ± 0.10%, respectively, compared with that of the LPS-treated group. Additionally, treatment with 5, 25, and 100 μg/mL of *I. britannica* extract encapsulated in liposomes reduced the expression of IL-1β by 13.23 ± 4.81%, 42.82 ± 1.58%, and 62.79 ± 1.29%, respectively, compared with that of the LPS-treated group.

### 3.5. Effect of I. britannica Extract Encapsulated in Liposomes on NF-κB Activation in LPS-Induced RAW 264.7 Cells

**Figure 4:**
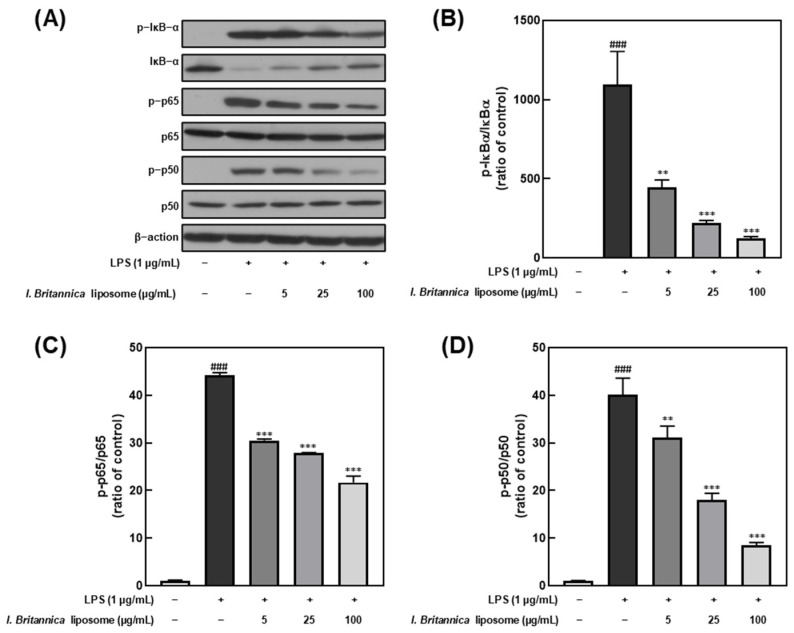
Comparative analysis of NF-κB protein expression level following *I. britannica* extract encapsulated in liposomes treatment in LPS-induced RAW 264.7 cells. (**A**) Western blot comparison of protein expression. Quantitative comparative analysis of phosphorylation of (**B**) IκB-α, (**C**) p-65, and (**D**) p-50. Data are represented as mean ± SEM (error bar). ^###^ *p* < 0.001 versus non-LPS-induced cells., ** *p* < 0.01, and *** *p* < 0.001 versus LPS-only treated cells. The data were statistically analyzed using a one-way ANOVA followed by Tukey’s post hoc test.

NF-κB is a transcription factor that plays a critical role in regulating immune and inflammatory responses upon stimulation by various factors, including LPS [32]. Furthermore, NF-κB activation results in the transcription of genes encoding pro-inflammatory cytokines and other inflammatory mediators. In this study, we investigated the effect of *I. britannica* extract encapsulated in liposomes on NF-κB activation in LPS-stimulated RAW 264.7 cells by treating them with various extract concentrations and examining the phosphorylation levels of IκB-α, p65, and p50. We found that IκB-α, p65, and p50 phosphorylation levels dose-dependently decreased upon treatment with *I. britannica* extract encapsulated in liposomes (Figure 4). Notably, at a 100 μg/mL concentration, we observed significant reductions in phosphorylation levels, including 88.61 ± 0.79% for IκB-α, 50.89 ± 2.50% for p65, and 78.72 ± 1.18% for p50, compared with that of the LPS-treated group.

### 3.6. Effect of I. britannica Extract Encapsulated in Liposomes on the Phosphorylation of MAPKs in LPS-Induced RAW 264.7 Cells

**Figure 5:**
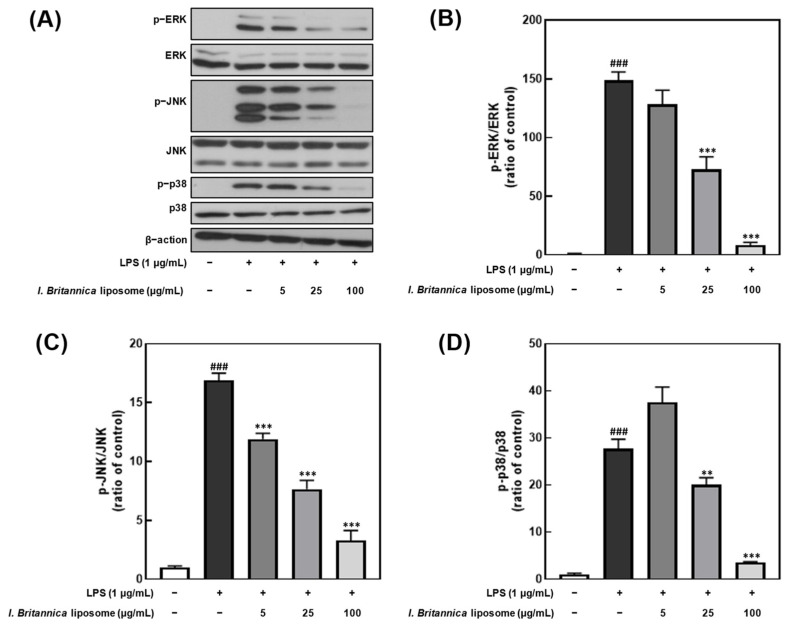
Comparative analysis of mitogen-activated protein kinase (MAPK) protein expression level following *I. britannica* extract encapsulated in liposomes treatment in lipopolysaccharide (LPS)-induced RAW 264.7 cells. (**A**) Western blot comparison of protein expression. Quantitative comparative analysis of phosphorylation of (**B**) ERK, (**C**) JNK, and (**D**) p-38. Data are represented as mean ± SEM (error bar). ^###^ *p* < 0.001 versus non-LPS-induced cells. ** *p* < 0.01, and *** *p* < 0.001 versus LPS-only treated cells. The data were statistically analyzed using a one-way ANOVA followed by Tukey’s post hoc test.

To investigate whether the inhibitory effect of *I. britannica* extract encapsulated in liposomes on NO production was associated with the inhibition of extracellular signal-regulated kinase (ERK), Jun N-terminal kinase (JNK), and p38 phosphorylation, we treated LPS-stimulated RAW 264.7 cells with various concentrations of *I. britannica* extract encapsulated in liposomes and measured the phosphorylation levels of ERK, JNK, and p38 (Figure 5). We found that the phosphorylation of ERK, JNK, and p38 decreased in a dose-dependent manner on treatment with *I. britannica* extract encapsulated in liposomes, and significant inhibitory effects (93.42 ± 1.41%, 72.38 ± 5.79%, and 87.22 ± 0.49%) were observed at a concentration of 100 μg/mL compared with that of the LPS-treated group (*p* < 0.001).

## 4. Discussion

In this study, the bioavailability of *I. britannica* extract was enhanced by encapsulating it in liposomes using a liposome preparation method that utilizes non-halogenated solvents such as ethyl acetate and *n*-hexane. This approach overcomes the disadvantages of traditional methods that use halogenated solvents, which are known to cause nerve, liver, and kidney damage and are difficult to remove from the production process [33]. By using non-halogenated solvents, our liposome preparation method offers several advantages, including reduced toxicity, no risk of nerve damage or harm to vital organs, and a simpler and more cost-effective production process.

Liposomes encapsulating *I. britannica* extract exhibited a uniform spherical morphology with a diameter of approximately 100 nm, as depicted in Figure 1. The zeta potential, which indicates the stability of the nanoparticles, was found to be −42.2 ± 0.7 mV, indicating a stable nanoparticle form of the liposomes. Zeta potential is a measure of the electrostatic repulsion between particles in a suspension and is a crucial factor in nanoparticle stability [34]. When the zeta potential value is high and negative or positive, the electrostatic repulsion between the nanoparticles is increased, leading to improved stability. A zeta potential value between −30 and −50 mV generally provides a stable nanoparticle suspension, as is observed in *I. britannica* extract encapsulated in liposomes. Furthermore, the encapsulation efficiency of *I. britannica* extract encapsulated in liposomes was 17.28 ± 0.39%, which is a typical value for liposomes containing water-soluble compounds. However, the encapsulation efficiency of water-soluble compounds in liposomes is generally lower than that of lipophilic compounds owing to the limitations of the W/O/W emulsion method used to prepare liposomes. While lipophilic compounds are typically incorporated into the lipid bilayer structure, resulting in high encapsulation efficiency, water-soluble compounds have a lower encapsulation efficiency, typically around 20% [35].

A comparative analysis of toxicity and anti-inflammatory activity (NO production) between *I. britannica* extract and *I. britannica* extract encapsulated in liposomes was performed to evaluate the potential of liposomes as a delivery system for the extract (Figure S2). We found that *I. britannica* extract encapsulated in liposomes was non-cytotoxic and exhibited higher anti-inflammatory activity compared with that of the same concentration of free extract. This can be attributed to the enhanced bioavailability and targeted delivery of the extract that were facilitated by the liposome system. The increased cellular uptake rate could be associated with the phospholipid bilayer structure of the liposome, which can interact with and fuse to the cell membrane, facilitating the delivery of the encapsulated extract into the cells [36].

Oxidative stress, resulting from an imbalance between reactive oxygen/nitrogen species (ROS/RNS) and the body’s antioxidant defenses, plays a significant role in inflammation and a range of diseases [37]. This imbalance mainly occurs in the mitochondria, where most ROS are generated during the electron transport chain process [38]. The overproduction of ROS can lead to the generation of harmful species such as superoxide anions, hydrogen peroxide, and hydroxyl radicals, which can interact with NO to produce damaging molecules like peroxynitrite [38]. This crosstalk between ROS and NO can amplify oxidative stress and inflammation, contributing to disease conditions including Parkinson’s disease, alcohol-induced liver damage, and other metabolic disorders [23,39]. Consequently, the regulation of ROS and NO levels, in concert with the enhancement of the body’s antioxidant defense systems, is crucial in the management of inflammation and oxidative stress-related diseases.

Herein, we investigated the anti-inflammatory effects of *I. britannica* extract encapsulated in liposomes on LPS-activated RAW 264.7 macrophages, highlighting the intricate interplay between inflammatory responses and oxidative reactions within cells. This interaction involves various signaling molecules, notably NO and PGE_2_. Generally, NO, which plays a role in blood pressure regulation, cell signaling, antibacterial action, and immune cell activation, is one of the most crucial signaling molecules generated within cells, and its levels significantly increase during inflammation and oxidative stress [40]. While small amounts of NO aid in cell protection and regulation of inflammatory responses, excessive amounts of NO can exacerbate oxidative stress, leading to DNA damage, inflammation amplification, cell necrosis, functional impairment, and increased vascular permeability [41]. Furthermore, NO production during inflammation is associated with the synthesis of the iNOS enzyme [42]. Therefore, we assessed the expression levels of iNOS transcription factors. Notably, PGE_2_, ultimately produced in immune responses, is responsible for vasodilation, pain induction, fever generation, and immune cell activation. It is synthesized by COX-2 when arachidonic acid signaling is disrupted by factors such as TNF-α, NO, heavy metals, UV radiation, oxidative stress, and injury [43]. Overproduction of PGE_2_ can worsen inflammation and foster the onset of diseases. To demonstrate the anti-inflammatory properties of *I. britannica* extract encapsulated in liposomes, we confirmed the suppression of NO production and PGE_2_ synthesis and the downregulation of iNOS and COX-2 transcription factors that are involved in NO and PGE_2_ production. Our results indicated that the *I. britannica* extract encapsulated in liposomes led to a concentration-dependent decrease in NO and PGE_2_ production. Hence, the regulation of NO and PGE_2_ may be crucial in preventing and treating inflammation and oxidative stress-related diseases.

Furthermore, we observed that the *I. britannica* extract encapsulated in liposomes effectively suppressed the expression of the pro-inflammatory cytokines TNF-α, IL-6, and IL-1β. In LPS-stimulated RAW 264.7 cells, pro-inflammatory cytokines play a crucial role in regulating various immune and inflammatory responses within the body [44]. When macrophages are stimulated by LPS, they produce and secrete TNF-α, which, along with LPS, induces the production of IL-6 and IL-1β. Notably, TNF-α is involved in various physiological processes, including inflammation and cellular injury [45]. Furthermore, IL-6, an essential pro-inflammatory cytokine produced by macrophages, contributes to acute immune responses, while IL-1β plays a vital role in initiating and intensifying inflammatory responses to bacterial infections [46,47]. Our findings demonstrated that *I. britannica* extract encapsulated in liposomes reduced the expression levels of pro-inflammatory cytokines such as TNF-α, IL-6, and IL-1β in a concentration-dependent manner. This finding suggests that the liposomes containing *I. britannica* extract suppress inflammation-related factors, by particularly inhibiting the initial stages of the inflammatory response.

As a final elucidation of the anti-inflammatory mechanisms, we investigated the effects of *I. britannica* extract encapsulated in liposomes on RAW 264.7 macrophages by examining their influence on key molecular components of the NF-κB and MAPK pathways. As previously mentioned, the *I. britannica* extract encapsulated in liposomes effectively suppressed the expression of the pro-inflammatory cytokines TNF-α, IL-6, and IL-1β. To further understand the underlying mechanisms, we focused on the regulation of NF-κB-related factors p50, p65, and IκB-α and MAPK-related factors ERK, JNK, and p38. Furthermore, the NF-κB pathway is a critical regulator of inflammation, and its activation is triggered by pro-inflammatory cytokines such as TNF-α. In unstimulated cells, the NF-κB dimer, which typically consists of p50 and p65 subunits, is bound to the inhibitory protein IκB-α and remains inactive in the cytoplasm [48]. Upon stimulation, IκB-α is phosphorylated and degraded, resulting in the release and activation of the p50–p65 dimer, which then translocates to the nucleus and initiates the transcription of target genes involved in inflammation [49]. We found that *I. britannica* extract encapsulated in liposomes effectively reduced the phosphorylation and degradation of IκB-α in a concentration-dependent manner, preventing the release and activation of the p50–p65 dimer. Additionally, we observed a concentration-dependent decrease in the nuclear translocation of p50 and p65 subunits, indicating the inhibition of NF-κB activation by *I. britannica* extract encapsulated in liposomes.

The MAPK signaling pathway, which involves ERK, JNK, and p38, plays a crucial role in cell growth, division, stress response, and cytokine regulation [50]. External stimuli, such as pro-inflammatory cytokines, activate the intracellular signal transduction pathway mediated by ERK, JNK, and p38, causing cell morphological changes and cytokine transcription [51,52]. The activation of p38, in particular, has been reported to induce the activity of p65, an NF-κB activator [53]. We observed that *I. britannica* extract encapsulated in liposomes reduced the phosphorylation levels of ERK, JNK, and p38 in a concentration-dependent manner, suggesting inhibition of the MAPK pathway. This modulation of MAPK signaling further supports the anti-inflammatory effects of *I. britannica* extract encapsulated in liposomes in RAW 264.7 macrophages.

## 5. Conclusions

Our study demonstrated the potential of *I. britannica* extract encapsulated in liposomes as a functional food ingredient with anti-inflammatory properties. By encapsulating the extract within liposomes using a safer and more cost-effective preparation method than the existing methods, we were able to enhance the bioavailability and targeted delivery of the extract. The *I. britannica* extract encapsulated in liposomes effectively suppressed the production of pro-inflammatory mediators, such as NO and PGE_2_, and downregulated the expression of iNOS and COX-2 transcription factors. Furthermore, the extract-containing liposomes reduced the production of pro-inflammatory cytokines TNF-α, IL-6, and IL-1β, and modulated the NF-κB and MAPK signaling pathways in LPS-stimulated RAW 264.7 macrophages. These findings suggest that *I. britannica* extract encapsulated in liposomes can serve as a valuable functional food ingredient to help manage and prevent inflammation-related disorders. The enhanced bioavailability, targeted delivery, and effective suppression of inflammatory mediators and cytokines make it a promising candidate for incorporation into various functional food products, such as supplements, beverages, or snacks, aimed at promoting health and well-being. Further studies on the efficacy of *I. britannica* extract encapsulated in liposomes using in vivo models and human clinical trials are warranted to confirm its safety and potential benefits in managing inflammation and associated diseases. Additionally, investigating the synergistic effects of *I. britannica* extract encapsulated in liposomes in combination with other anti-inflammatory compounds or food ingredients to develop innovative and effective functional food products is of significance.

## Figures and Tables

**Table 1:** Characterization of liposomes encapsulating *I. britannica* extract.

Diameter Size (nm)	Polydiversity (PdI)	Z-Potential (mV)
101.3 ± 5.6	0.012 ± 0.007	−42.2 ± 0.7

## Data Availability

Not applicable.

## Supplementary Materials

The following supporting information can be downloaded at: https://www.mdpi.com/article/10.3390/antiox12081636/s1, Figure S1: High-performance liquid chromatography (HPLC) analysis of chlorogenic acid for determining the encapsulation efficiency of *I. britannica* extract in liposomes. HPLC chromatogram of chlorogenic acid contents of standard compounds (A), *I. britannica* extract (B), and *I. britannica* extract encapsulated in liposomes (C). Figure S2: Evaluation of cytotoxicity and anti-inflammatory effects of *I. britannica* extract and *I. britannica* extract encapsulated in liposomes in LPS-stimulated RAW 264.7 cells. (A) cytotoxicity; (B) nitric oxide (NO) production measurement.
