# Supplementary material for: Development and Characterization of Inula britannica Extract-Loaded Liposomes: Potential as Anti-Inflammatory Functional Food Ingredients
Source: Antioxidants (Basel). 2023 Aug 18;12(8):1636. doi: 10.3390/antiox12081636 (PMC10451523; doi:10.3390/antiox12081636)
Supplement: Supplementary file 1 [file antioxidants-12-01636-s001.zip › antioxidants-2453732-supplementary.pdf]

Supplementary Data

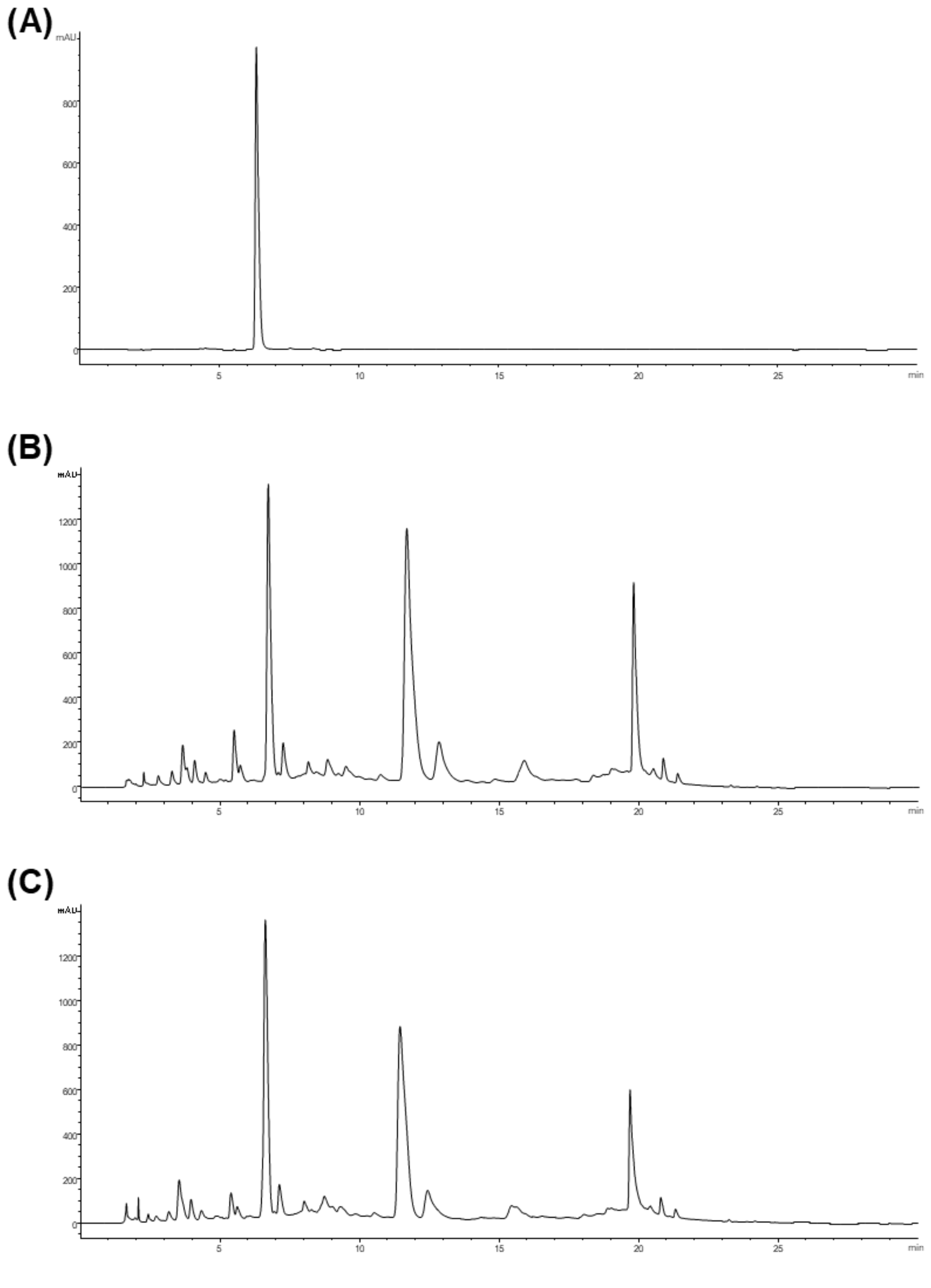

Figure S1. High-performance liquid chromatography (HPLC) analysis of chlorogenic acid for determining the encapsulation efficiency of *I. britannica* extract in liposome. HPLC chromatogram of chlorogenic acid contents of standard compound (**A**), *I. britannica* extract (**B**), *I. britannica* extract encapsulated in liposome (**C**).

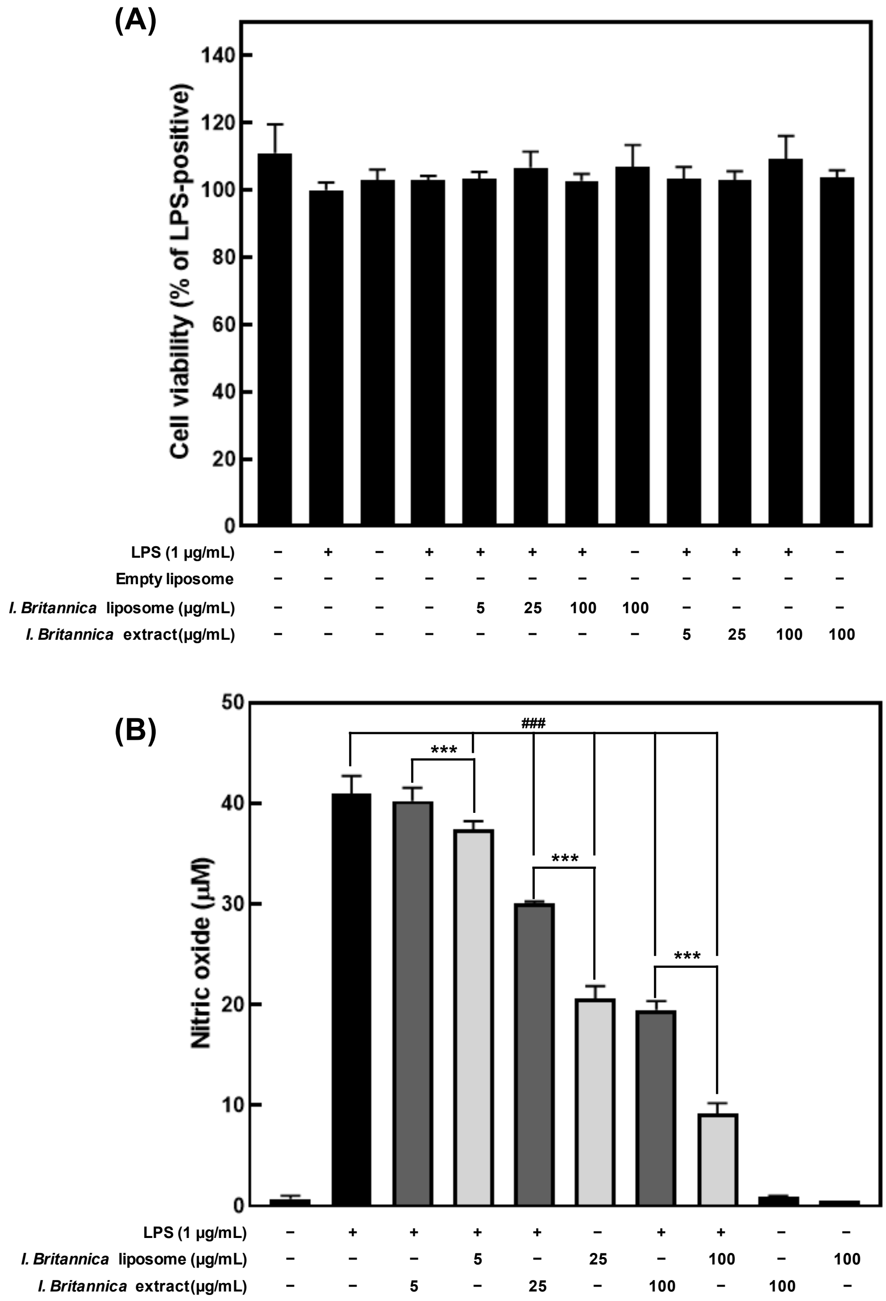

Figure S2. Evaluation of cytotoxicity and anti-inflammatory effects of *I. britannica* extract and *I. britannica* extract encapsulated in liposomes in LPS-stimulated RAW 264.7 cells. (**A**) Cytotoxicity, (**B**) nitric oxide (NO) production measurement. Data are represented as mean ± SEM (error bar). ###$p < 0.001$ versus to LPS-induced cells. ***$p < 0.001$ compared to both *I. britannic*a extract and *I. britannica* extract encapsulated in liposomes. Data were statistically analyzed using one-way ANOVA followed by Tukey's post hoc test.
